# MAKING THE MOST: UNDERSTANDING COMMUNITY IN SEXUAL- AND GENDER-DIVERSE COMMUNITIES

**DOI:** 10.1093/geroni/igae098.1205

**Published:** 2024-12-31

**Authors:** M Aaron Guest, Christine Happel, Erin Burk-Leaver

**Affiliations:** Chair; Co-Chair; Discussant

## Abstract

In program service and delivery, community significantly impacts the health and well-being of older adults, providing essential social support, fostering belonging, and ensuring access to vital resources and services. Beyond mere geographic proximity, communities embody shared traits, interests, values, and goals, aiding members in accessing various services and activities crucial for healthy aging. This includes fostering engagement, inclusivity, and support. Importantly, individuals often belong to multiple communities, enhancing their resource access as they age. A notable example is the sexual and gender-diverse community, comprising individuals identifying as gay, lesbian, bisexual, and transgender, among other identities. These individuals, especially older ones, face considerable barriers in accessing care, support, and resources, often lacking the opportunities available to their heterosexual counterparts. This symposium delves into how older individuals from these diverse backgrounds form and participate in communities. Happel et al.‘s work explores the unique experiences of loneliness, isolation, and service use among older LGBTQ adults. Guest et al. identify how community connection can affect self-perceptions of aging, resulting in accelerated social aging and adverse health outcomes. Mccoyy et al. then share a community-based participatory action approach to identifying and addressing the needs of sexual and gender-diverse older adults through alliance building. The shared theme of community and how SGD individuals create and use community will be highlighted through exemplars leading to a discussion of how we can create health-promoting communities for SGD individuals as they age.

